# Astaxanthin protects ARPE-19 cells from oxidative stress via upregulation of Nrf2-regulated phase II enzymes through activation of PI3K/Akt

**Published:** 2013-07-25

**Authors:** Zhongrui Li, Xin Dong, Hongling Liu, Xi Chen, Huanqi Shi, Yan Fan, Dingshan Hou, Xiaomei Zhang

**Affiliations:** 1 Department of Ophthalmology, The First Affiliated Hospital of Harbin Medical University, Harbin, Heilongjiang Province, P.R. China; 2 Department of Pharmacy, The First Affiliated Hospital of Harbin Medical University, Harbin, Heilongjiang Province, P.R. China

## Abstract

**Purpose:**

Oxidative stress on retinal pigment epithelial (RPE) cells is thought to play a crucial role in the development and progression of age-related macular degeneration. Astaxanthin (AST) is a carotenoid that shows significant antioxidant properties. This study was designed to investigate the protective effect of AST on ARPE-19 cells against oxidative stress and the possible underlying mechanism.

**Methods:**

ARPE-19 cells exposed to different doses of H_2_O_2_ were incubated with various concentrations of AST and cell viability subsequently detected with the (4-[3-[4-iodophenyl]-2–4(4-nitrophenyl)-2H-5- tetrazolio-1,3-benzene disulfonate]; WST-1) assay. The apoptosis rate and intracellular levels of reactive oxygen species (ROS) were measured with flow cytometry. NAD(P)H quinine oxidoreductase 1 (NQO1), hemeoxygenase-1 (HO-1), glutamate-cysteine ligase modiﬁer subunit (GCLM), and glutamate-cysteine ligase catalytic subunit (GCLC) expression were examined with real-time PCR and western blotting. The nuclear localization of nuclear factor (erythroid-derived 2)-like 2 (Nrf2) protein and the expression levels of cleaved caspase-3 and protein kinase B proteins were evaluated with western blotting.

**Results:**

AST clearly reduced H_2_O_2_-induced cell viability loss, cell apoptosis, and intracellular generation of ROS. Furthermore, treatment with AST activated the Nrf2-ARE pathway by inducing Nrf2 nuclear localization. Consequently, Phase II enzymes NQO1, HO-1, GCLM, and GCLC mRNA and proteins were increased. AST inhibited expression of H_2_O_2_-induced cleaved caspase-3 protein. Activation of the phosphatidylinositol 3-kinase/protein kinase B (PI3K/Akt) pathway was involved in the protective effect of AST on the ARPE-19 cells.

**Conclusions:**

AST protected ARPE-19 cells against H_2_O_2_-induced oxidative stress via Nrf2-mediated upregulation of the expression of Phase II enzymes involving the PI3K/Akt pathway.

## Introduction

Age-related macular degeneration (AMD) is a major cause of irreversible vision loss in elderly people in the developed world [1,2]. Although the pathogenic mechanism of AMD is poorly understood, recent studies have shown that oxidative stress has an important role in AMD pathogenesis [1]. Research demonstrates that pathologic damage to retinal pigment epithelial (RPE) cells is an early event in AMD, and the RPE cell is known to be a primary target in this condition [3,4]. Oxidative stress is thought to be particularly significant in the development of age-related RPE cell degeneration, dysfunction, and loss [3]. Therefore, recent studies have focused on methods for protecting RPE cells from oxidative stress to slow AMD [5,6].

**Figure 1:**
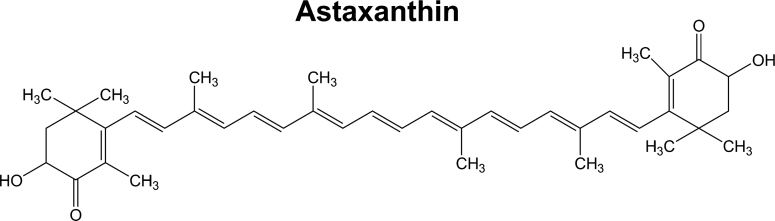
Chemical structure of astaxanthin. 3,3′-dihydroxy-β,β′-carotene-4,4′-dione.

Astaxanthin (3,3′-dihydroxy-β,β′-carotene-4,4′-dione, AST; Figure 1 shows the chemical structure) is a well-known non-provitamin A xanthophyll carotenoid of predominantly marine origin [7-9]. AST has been reported to possess a wide variety of biologic functions, including anti-inflammatory, antiapoptotic ,and anticarcinogenic activity, as well as neuroprotective and cardioprotective effects [7,8,10-12]. In addition to these activities, AST has been shown to have a high level of antioxidant activity: 10 times higher than that of other carotenoids, such as lutein, canthaxanthin, and β-carotene and 100 times higher than α-tocopherol [13]. Currently, many kinds of AST products are sold in the form of nutritional supplements [14]. Human clinical studies have used oral AST in a dose that varies from 4 mg up to 100 mg/day [7]. In a study conducted by Coral-Hinostroza et al., a maximum plasma concentration of 0.28±0.1 mg/l AST was observed in the first 11.5 h after administration, and the plasma astaxanthin elimination half-life was 52±40 h [15]. Furthermore, it was reported recently that the intake of antioxidants, including AST, might prevent AMD by improving visual acuity and function [16].

It is not known whether AST can protect the RPE cell against oxidative damage. In this study, we investigated the cytoprotective effect of astaxanthin on oxidative stress induced by H_2_O_2_ in ARPE-19 cells and explored the underlying mechanisms.

## Methods

### Materials

The human RPE cell line ARPE-19 was obtained from the American Type Culture Collection (ATCC, Mantissa, VA). Astaxanthin, 2′,7′-dichlorodihydroﬂuorescein diacetate (DCFH-DA), and LY294002 were purchased from Sigma (St. Louis, MO). The antibody for nuclear factor (erythroid-derived 2)-like 2 (Nrf2), NAD(P)H: quinine oxidoreductase 1 (NQO1), and Lamin B were obtained from Santa Cruz Biotechnology (Santa Cruz, CA). Antibodies for protein kinase B (Akt), p-Akt, and cleaved caspase-3 were purchased from CST Cell Signaling Technology (Watham, MA). Antibodies for hemeoxygenase-1 (HO-1), glutamate-cysteine ligase modiﬁer subunit (GCLM), and glutamate-cysteine ligase catalytic subunit (GCLC) were obtained from Abcam (Cambridge, MA). Dulbecco’s modiﬁed Eagle medium and fetal bovine serum were obtained from Gibco BRL (Grand Island, NY). 4-[3-[4-iodophenyl]-2–4(4-nitrophenyl)-2H-5- tetrazolio-1,3-benzene disulfonate] (WST-1) was obtained from Roche (Mannheim, Germany). The Annexin V fluorescein isothiocyanate (FITC)-Propidium Iodide (PI) apoptosis kit was purchased from Becton Dickinson (Mountain View, CA).

### Cell culture

ARPE-19 cells (from ATCC cell line) were cultured in Dulbecco’s modiﬁed Eagle medium supplemented with 10% (v/v) heat-inactivated fetal bovine serum, 100 μg/ml of streptomycin, and 100 U/ml of penicillin. The cells were cultured at 37 °C in a humidified 5% CO_2_ atmosphere, and the medium was changed every other day. The cells were grown to an appropriate density and used at passage 10–15.

### Measurement of cell viability

Cell viability was determined with the sulfonated tetrazolium salt WST-1. The measurement depends on the ability of viable cells to cleave tetrazolium salts by mitochondrial dehydrogenases. Briefly, cells were plated in 96-well microplates at a density of 5×10^4^ cells/well. After incubation for 24 h at 37 °C, cells were treated with 0, 5, 10, or 20 µM AST for 6 h, 12 h, and 24 h at 37 °C. The cells were then treated with 200 or 400 μM H_2_O_2_ for 24 h at 37 °C, WST-1 solution was added (10 µl/well), and the cells were further incubated for 3 h at 37 °C in a 5% (v/v) CO_2_ atmosphere. Absorbance at 450 nm was measured with a microplate reader with a background control as the blank.

### Flow cytometry analysis of cell apoptosis

The Annexin V FITC-Propidium Iodide (PI) kit was used to detect cell apoptosis. The cells were grown on a six-well plate at 1×10^5^ cells/well and treated with or without different concentrations of AST for 24 h at 37 °C, before treatment with 200 μM H_2_O_2_ for 24 h at 37 °C. The cells were washed twice and collected with PBS (137 mM NaCl, 2.7 mM KCl, 4.3 mM Na_2_HPO_4_, 1.4 mM KH_2_PO_4_). Staining for apoptosis was done according to the manufacturer’s instructions. PI-negative, Annexin V-negative staining cells are considered live cells; PI-negative, Annexin V-positive staining cells are considered early apoptotic cells. The stained cells were analyzed with FACSCalibur flow cytometry (BD Biosciences, San Jose, CA) with Cell-Quest software.

### Measurement of accumulation of intracellular reactive oxygen species

The intracellular levels of reactive oxygen species (ROS) were measured using the DCFH-DA molecular probes. Cells were incubated with 10 μM DCFH-DA for 30 min at 37 °C, then washed, and resuspended in PBS at 1×10^6^ cells/ml. The cells were analyzed using flow cytometry at excitation and emission wavelengths of 488 and 525 nm, respectively. Untreated cells served as the control. The results were expressed as fluorescence intensity of dichlorofluorescein (DCF) compared with control.

### Real-time polymerase chain reaction

Total RNA was extracted from the cells using TRIzol reagent (Invitrogen, Carlsbad, CA) as described by the manufacturer. Samples containing 1 μg of total RNA were reverse transcribed into cDNA with a ﬁrst-strand cDNA synthesis kit (Bio-Rad, Hercules, CA) according to the manufacturer’s instructions. Real-time PCR was performed with the SYBR Green PCR Master Mix kit (Applied Biosystems, Foster City, CA) on a Bio-Rad iCycler system (Bio-Rad). The fold change in the levels of NQO1, GCLM, HO-1, and GCLC between the treated and untreated cells, normalized by the level of β-actin, was determined using the following equation: fold change=2^−∆(∆Ct)^, where ∆Ct=Ct_(target)_ − Ct_(β-actin)_ and ∆(∆Ct)=∆Ct_(treated)_ − ∆Ct_(untreated)_. The primers used in this study were purchased from Invitrogen (Shanghai, China; Table 1).

**Table 1:** Primers for real-time PCR assay.

Gene	Primer (5′-3′)
NQO1	F: TATCCTGCCGAGTCTGTTCTG
	R: AACTGGAATATCACAAGGTCTGC
GCLM	F: ACTGACTTAGGAGCATAACTTACC
	R: AAGAATATCTGCCTCAATGACACC
HO-1	F: ATGACACCAAGGACCAGAGC
	R: GTAAGGACCCATCGGAGAAGC
GCLC	F: AAGCCATTCACTCCAGATTTTACC
	R: ACAACAAACTTCAACGCAAAGC
β-actin	F: TCGTGCGTGACATTAAGGAGAAG
	R: GTTGAAGGTAGTTTCGTGGATGC

### Western blot analysis

After treatments, the cells were twice washed gently in ice-cold PBS and then lysed using a Nuclear and Cytoplasmic Protein Extraction Kit (Beyotime, Haimen, China) according to the protocol described by the manufacturer. Lysates were centrifuged at 15,000 ×*g* for 10 min at 4 °C. Protein concentrations were determined with the Bicinchoninic Acid Protein Assay kit (Pierce, IL). Protein samples were fractionated with SDS-PAGE and then transferred to polyvinylidene diﬂuoride membranes (Millipore, Bedford, MA). After blocking with 5% (v/v) skim milk for 1 h at room temperature, membranes were incubated with primary antibodies overnight at 4 °C and then incubated with the corresponding horseradish peroxidase-linked secondary antibodies for 1 h at room temperature. The signals were developed using the enhanced chemiluminescence (ECL) western blotting detection reagent (Amersham Biosciences, Piscataway, NJ) and exposed to X-ray ﬁlm. Densitometric analysis was performed with Quantity One software (Bio-Rad Laboratories).

### Statistical analysis

Data were expressed as mean ± standard deviation (SD). All data were analyzed with one-way analysis of variance (ANOVA), followed by the Student–Newman–Keuls test for multiple comparisons. Statistical significance was set at p≤0.05.

## Results

### Astaxanthin prevents hydrogen peroxide–induced decrease in ARPE-19 cell viability

**Figure 2:**
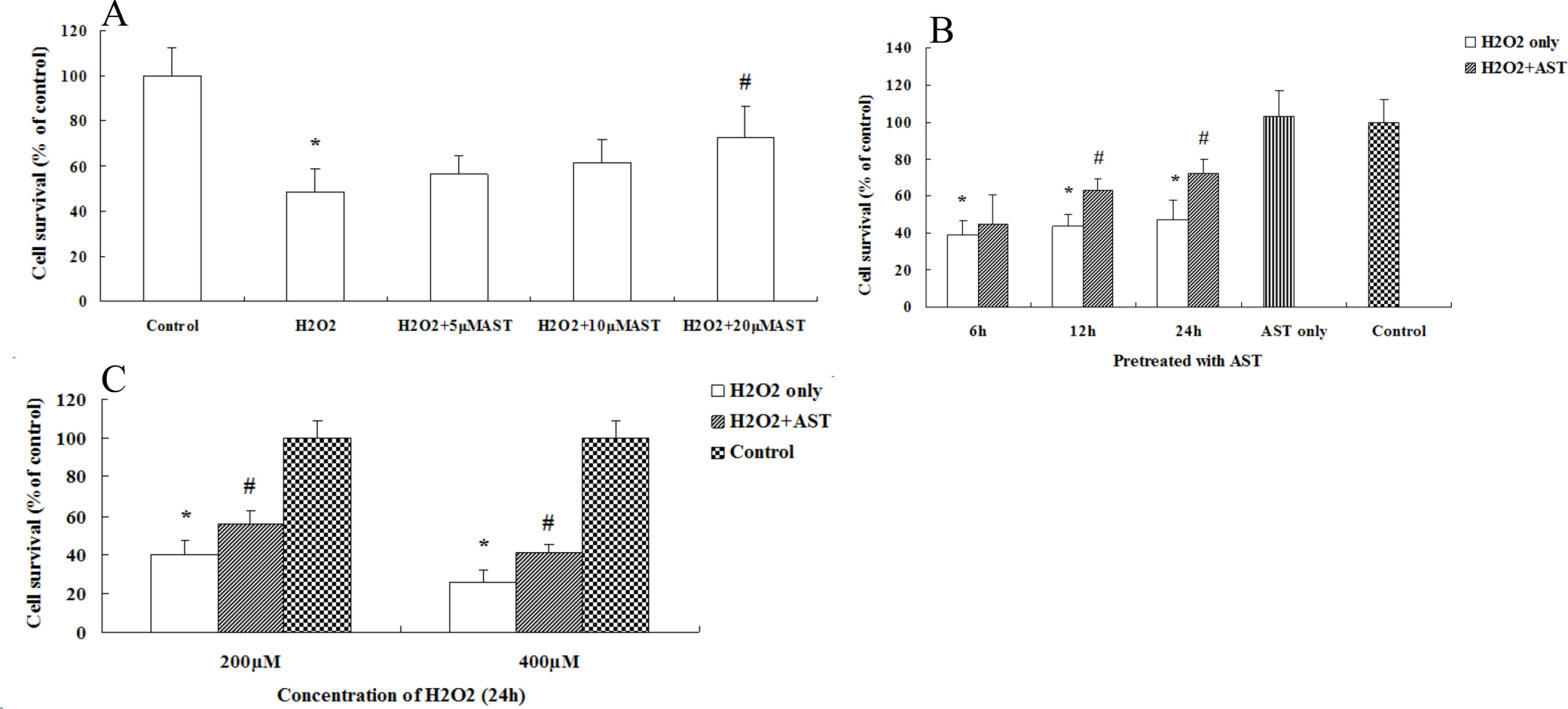
Astaxanthin prevented the decrease in retinal pigment epithelial cell viability induced by hydrogen peroxide. **A**: The ARPE-19 cells were incubated with different concentrations of astaxanthin (AST; 0, 5, 10, and 20 µM) for 24 h and then exposed to 200 µM hydrogen peroxide (H_2_O_2_) for 24 h. **B**: The ARPE-19 cells were treated with 20 µM AST for different lengths of time (6, 12, and 24 h) and then exposed to 200 µM H_2_O_2_ for 24 h. **C**: The ARPE-19 cells were treated with 20 µM AST for 24 h and then exposed to different concentrations of H_2_O_2_ (200 and 400 µM) for 24 h. Data are shown as mean ± standard deviation (SD) (n=6); *p<0.05 versus control. In all cases, the control is untreated retinal pigment epithelial (RPE) cells. # p<0.05 versus H_2_O_2_-induced cells without treatment with AST.

Oxidative damage to cells is commonly modeled using treatment with H_2_O_2_ [17-19]. Cell viability was evaluated using WST-1 assays. We first incubated ARPE-19 cells for 24 h with different concentrations of AST and then exposed the cells to 200 µM H_2_O_2_ for 24 h. Figure 2A shows a significant dose-dependent increase in cell viability; 20 µM was determined as the optimal dose for treatment with AST. To investigate whether the protective effect of AST is related to incubation time, we incubated ARPE-19 cells with AST for different lengths of time before the cells were exposed to H_2_O_2_. Figure 2B shows the cell viability increase was time-dependent and 24 h was the optimal time for AST treatment. Thus, we selected treatment with 20 µM AST for 24 h before exposure to different concentrations of H_2_O_2_. Figure 2C shows the protective effect of AST still existed when the concentration of H_2_O_2_ was 400 µM; 200 µM H_2_O_2_ caused an approximate 50% loss of cell viability without treatment with AST.

### Protective effect of astaxanthin against hydrogen peroxide–induced cell apoptosis

**Figure 3:**
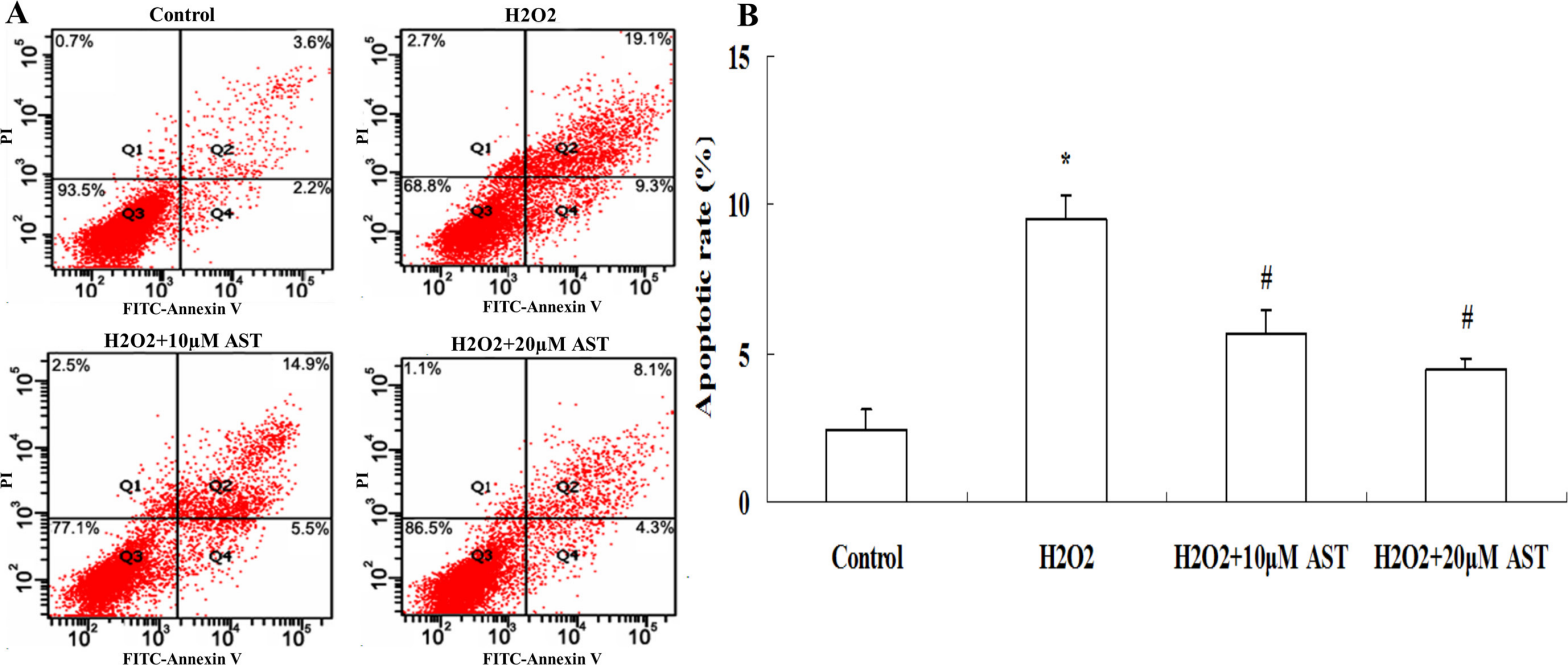
Astaxanthin inhibited H_2_O_2_-induced apoptosis in ARPE-19 cells. **A**: The ARPE-19 cells were incubated with 10 µM and 20 µM astaxanthin (AST) for 24 h and then exposed to 200 µM hydrogen peroxide (H_2_O_2_) for 24 h. Flow cytometry recording shows the apoptosis rate of the ARPE-19 cells. **B**: Summarized data show the rate of apoptotic cells detected with ﬂow cytometry. Data are shown as mean ± standard deviation (SD) (n=6); *p<0.05 versus control; # p<0.05 versus H_2_O_2_-induced cells without treatment with AST.

H_2_O_2_ induces cellular apoptosis. To investigate whether AST protects against H_2_O_2_-induced apoptosis, ARPE-19 cells were incubated with 10 μM and 20 μΜ AST for 24 h and then exposed to 200 µM H_2_O_2_ for 24 h. Cells were then stained with Annexin V⁄ PI, and the apoptosis rate was determined with flow cytometry. As shown in Figure 3, the lower right field (PI-negative, Annexin V-positive staining) indicates the apoptotic cells. Figure 3 shows a significant increase in the apoptosis rate when the ARPE-19 cells were exposed to 200 µM H_2_O_2_. Moreover, when the ARPE-19 cells were treated with different concentrations of AST, the cell attenuation of apoptosis was dose-dependent.

### Astaxanthin inhibits hydrogen peroxide–induced intracellular generation of reactive oxygen species

**Figure 4:**
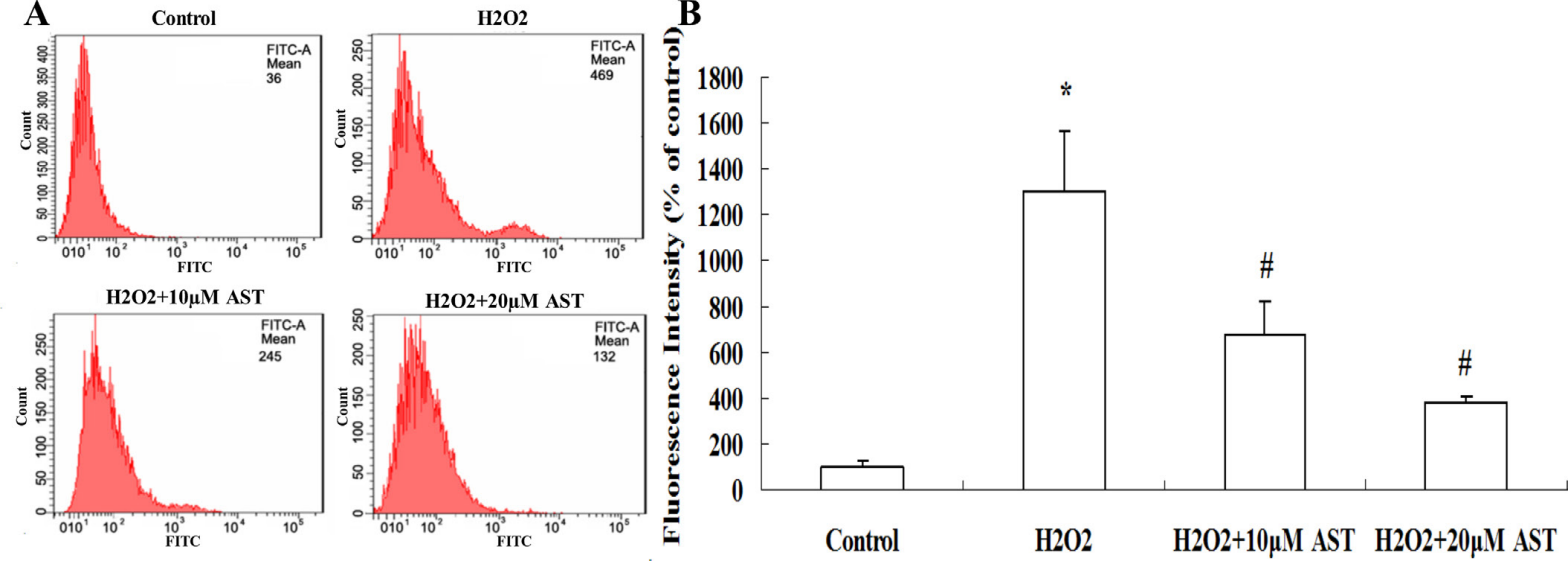
Astaxanthin inhibited hydrogen peroxide–induced intracellular generation of reactive oxygen species. **A**: The ARPE-19 cells were incubated with 10µM and 20µM astaxanthin (AST) for 24 h and then exposed to 200 µM hydrogen peroxide (H_2_O_2_) for 24 h. The intracellular reactive oxygen species (ROS) was measured with flow cytometry using 2′,7′-dichlorodihydroﬂuorescein diacetate (DCFH-DA). **B**: AST reduced the generation of ROS in ARPE-19 cells significantly. Data are shown as mean ± standard deviation (SD) (n=6); *p<0.05 versus control; # p<0.05 versus H_2_O_2_-induced cells without treatment with AST.

The ARPE-19 cells were incubated with 10 μM and 20 μΜ AST for 24 h and then treated with 200 µM H_2_O_2_ for 24 h. DCF fluorescence was recorded as a measure of intracellular ROS. Figure 4 shows the levels of intracellular ROS were increased significantly in the H_2_O_2_-treated cells. However, treatment with AST resulted in a dose-dependent inhibition of intracellular production of ROS.

### Effects of astaxanthin on the expression of NQO1, HO-1, GCLC, and GCLM mRNA and protein

**Figure 5:**
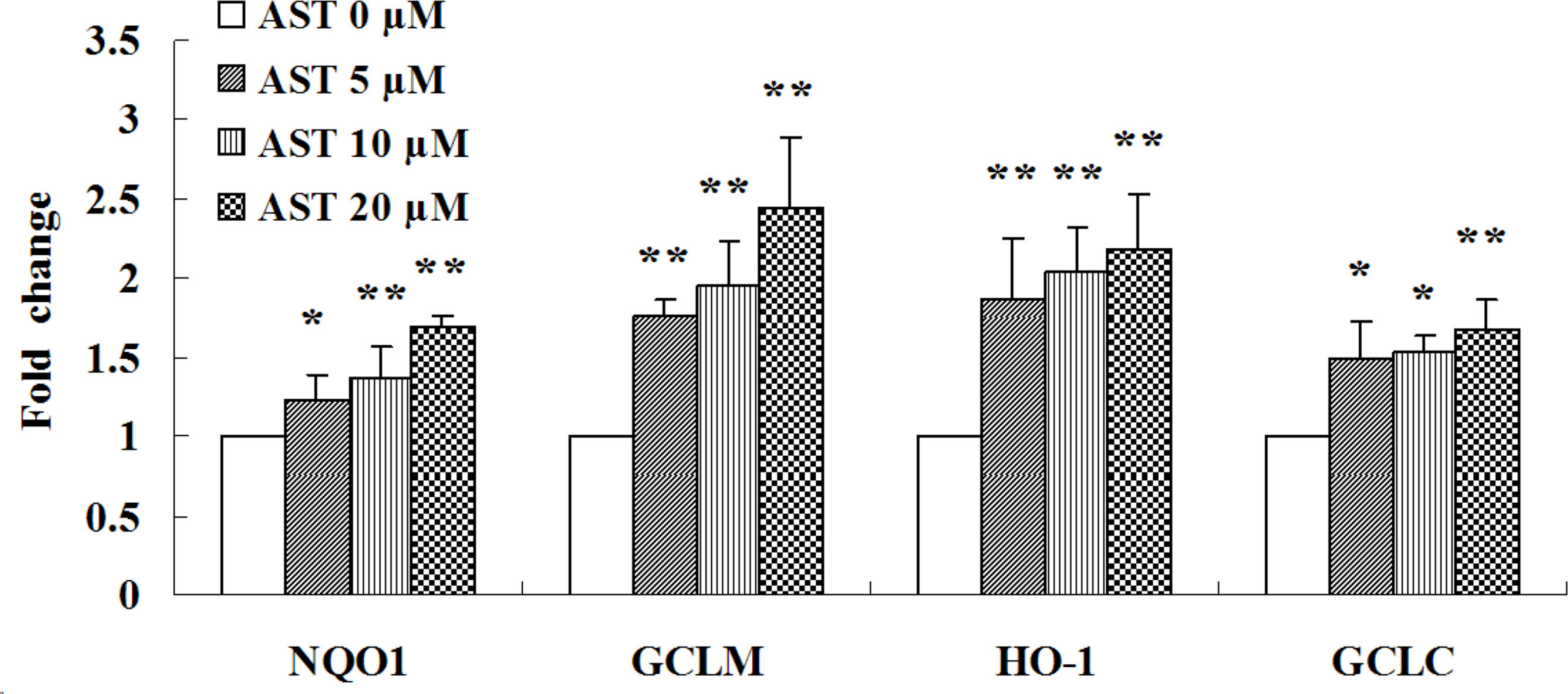
Astaxanthin increased expression of NQO1, GCLM, HO-1 and GCLC mRNA. Cells were treated with 5, 10, and 20 µM astaxanthin (AST) for 24 h. Total RNA was extracted, and the amounts of NQO1, GCLM, HO-1 and GCLC mRNA amounts were quantified with real-time PCR and normalized to the corresponding amounts of β-actin mRNA. Data are shown as mean ± standard deviation (SD) (n=6); *p<0.05 versus control; **p<0.01 versus control.

**Figure 6:**
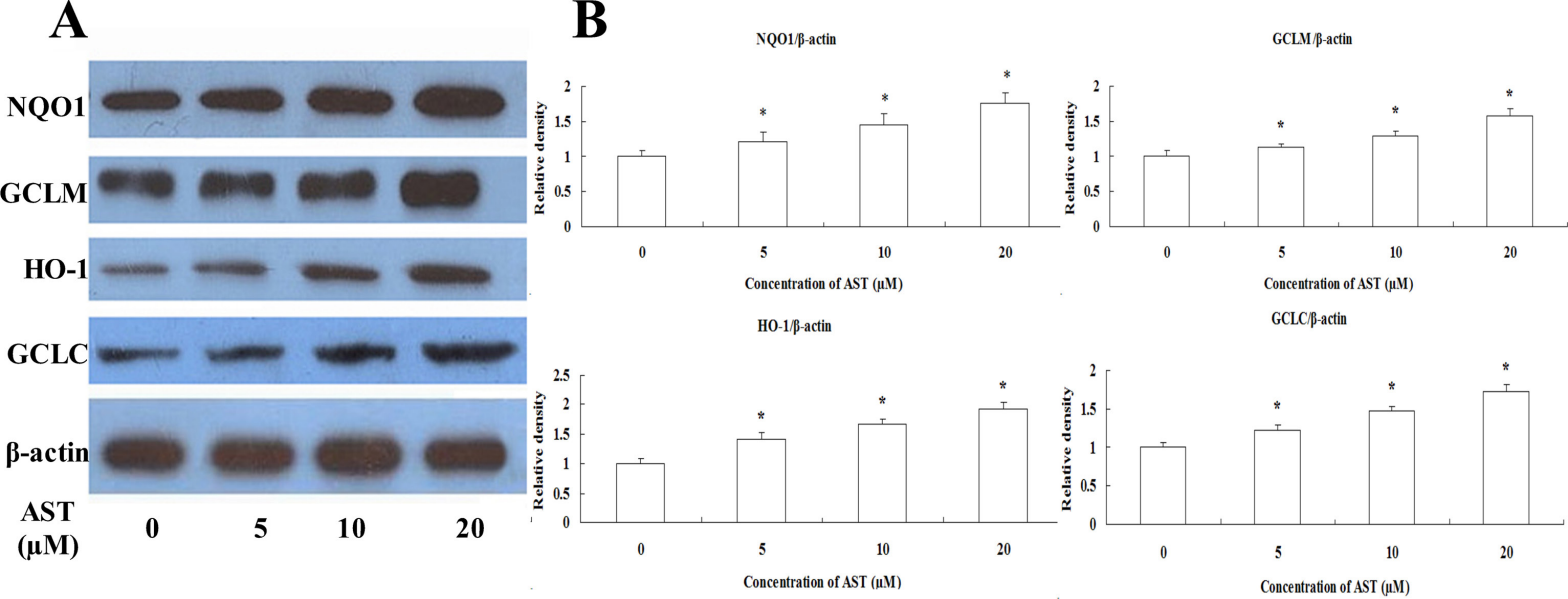
Astaxanthin increased expression of NQO1, GCLM, HO-1 and GCLC protein. **A**: Cells were treated with 5, 10, and 20 µM astaxanthin (AST) for 24 h. Western blot analysis was performed using the corresponding antibodies. **B**: Quantitative analysis of the relative protein levels in the ARPE-19 cells. Data are shown as mean ± standard deviation (SD) (n=6); *p<0.05 versus control.

To clarify the antioxidative mechanisms of AST against H_2_O_2_-induced cell injury in ARPE-19 cells, we examined the expression levels of Phase II enzymes, such as NQO1, HO-1, GCLM, and GCLC. Figure 5 shows treatment of ARPE-19 cells with different concentration of AST (5, 10, or 20 µM) for 24 h induced an increase in the expression of NQO1, HO-1, GCLC, and GCLM mRNAs. To expand these findings, we used western blotting to detect whether AST can induce the expression of Phase II enzymes. Consistent with the detection of mRNA encoding the Phase II enzymes (Figure 6), all the indicated concentrations of AST (5, 10, or 20 µM) resulted in enhancements of expressions of NQO1, HO-1, GCLC, and GCLM proteins. These results suggest that NQO1, HO-1, GCLC, and GCLM have important roles in the protective action of AST.

### Involvement of the PI3K/Akt pathway in astaxanthin-induced cytoprotection against oxidative stress

**Figure 7:**
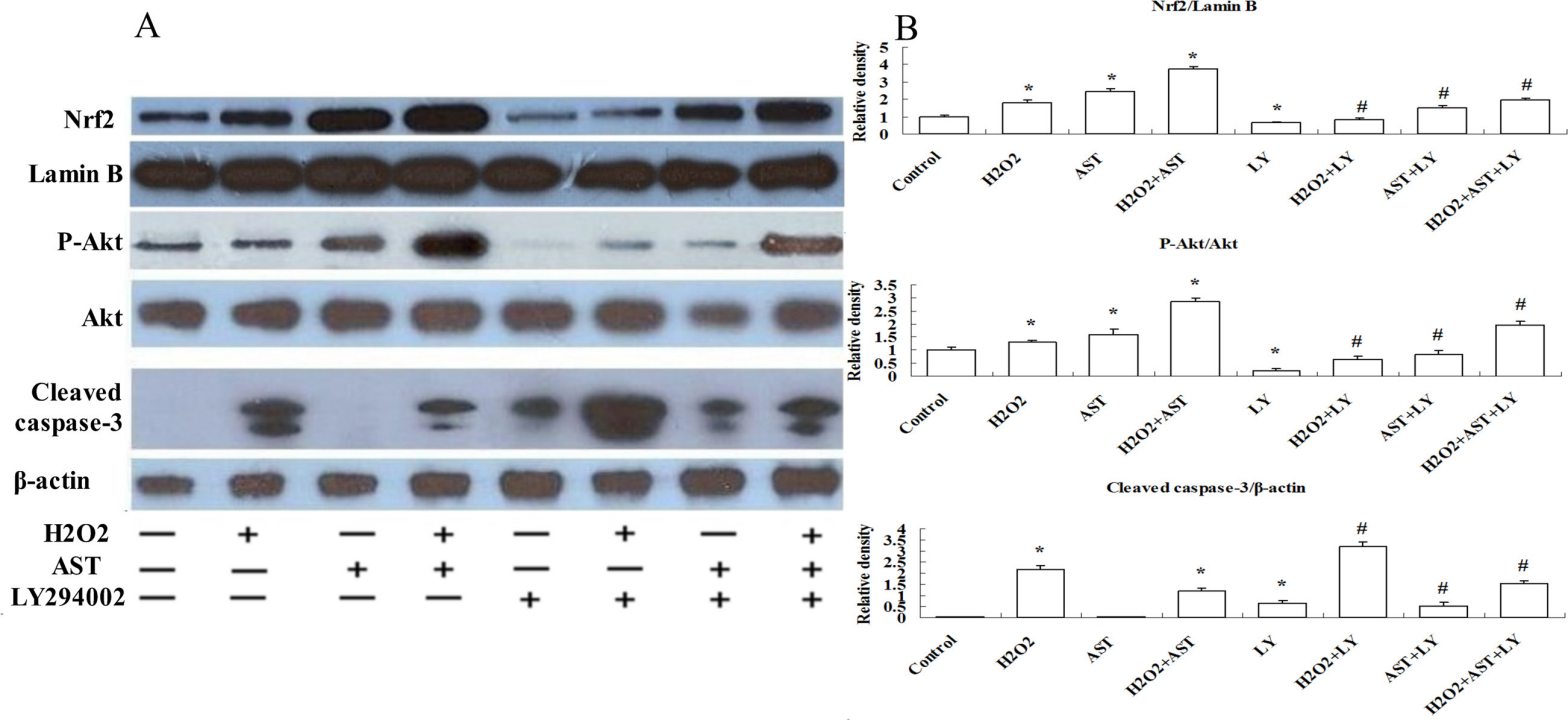
Activation of the PI3K/Akt pathway was involved in the protective effect of astaxanthin on ARPE-19 cells. **A**: Cells were treated with or without 20 µM astaxanthin (AST) for 24 h and then treated with or without 10 µM LY294002 for 30 min before incubation with or without 200 µM hydrogen peroxide (H_2_O_2_) for 24 h. Western blot analysis was done using the corresponding antibodies. Cytosolic fractions were immunoblotted with anti-protein kinase B (anti-Akt), anti-p-Akt, and anti-cleaved caspase-3 antibodies. Nuclear fractions were immunoblotted with anti-Nrf2 and anti-Lamin B antibodies. **B**: Quantitative analysis of the relative protein levels in ARPE-19 cells. Data are shown as mean ± standard deviation (SD) (n=6); *p<0.05 versus control; # p<0.05 versus H_2_O_2_ + AST.

The PI3K/Akt pathway has been reported to be essential in regulating the antioxidant function in the RPE cell [20]. Research indicated that Akt activation could protect RPE cells from oxidant-induced cell death [21]. It has been reported that AST could induce significant activation of PI3K in neural progenitor cells [22]. We examined Akt phosphorylation in ARPE-19 cells to determine whether the PI3K/Akt pathway is responsible for the AST-induced protective effect against oxidative stress. ARPE-19 cells were incubated with 20 µM AST for 24 h and then treated with 200 µM H_2_O_2_ for 24 h. Figure 7 shows a western blot assay indicating that treatment with AST before H_2_O_2_ markedly increased the level of expression of p-Akt (the activated form of Akt). However, coapplication of the specific inhibitor of Akt phosphorylation LY294002 (10 µM) abolished the AST-induced increase in the expression of p-Akt. These results suggest that AST is dependent on the activation of Akt to afford protection.

### Nuclear localization of Nrf2 protein induced by astaxanthin is regulated by the PI3K/Akt pathway

Because Nrf2 is a crucial transcription factor for regulating the expression of endogenous antioxidant enzymes, we asked whether AST could induce Nrf2 localization in ARPE-19 cells. Figure 7 shows the results of a western blot assay indicating that treatment with 20 µM AST increased the nuclear localization of Nrf2 significantly. This increased nuclear localization of Nrf2 was blocked by LY294002 (10 µM). These results suggest AST increases the nuclear localization of Nrf2 through the PI3K/Akt pathway.

### Downregulation of cleaved caspase-3 protein expression induced by astaxanthin is regulated by the PI3K/Akt pathway

We examined the protein expression of cleaved caspase-3 to investigate the underlying mechanism of the antiapoptotic effect of AST. Figure 7 shows treatment with 200 µM H_2_O_2_ markedly increased the cleavage of caspase-3. However, the increase was suppressed significantly by treatment with 20 µM AST. LY294002 (10 µM) partially reduced the AST-induced protective effect. These results reveal the involvement of the Akt pathway in the protective effect of AST on H_2_O_2_-induced apoptosis.

## Discussion

This study investigated the protective effect of AST on H_2_O_2_-induced oxidative stress in ARPE-19 cells and the possible signal pathway involved. AST has been shown to have significant antihypertensive, neuroprotective, anti-diabetes, and anti-obesity effects in experimental animals in vivo [23-25]. It has been reported that administration of AST could increase the choroidal blood flow velocity in volunteers [26]. Research indicated that supplementation with a formulation containing AST may improve visual function and help to delay progression of AMD [16]. AST is a powerful free radical scavenger and protects several types of cells from oxidative damage [8,27,28]. Although earlier studies showed that AST supplement leads to a decrease in the level of the TNF-α-induced MCP-1 protein in ARPE-19 cells, it is not known whether AST can protect ARPE-19 cells from oxidative stress [29]. Our results suggest that treatment with AST reduces H_2_O_2_-induced cell death, intracellular ROS production, and apoptosis in ARPE-19 cells, and that the mechanism by which AST induced cytoprotection could include the Nrf2-antioxidant-response element (ARE) and Akt pathways. These data indicate that AST might provide a valuable therapeutic strategy for early AMD.

Oxidative stress has been studied extensively in relation to the pathophysiology of AMD and is suggested to have a crucial role [1]. The location and physiologic function of RPE cells cause them to be constantly exposed to several ROS [3,30]. Thus, protecting RPE cells from oxidative damage is an important consideration for treating AMD. The addition of H_2_O_2_ to cultured cells is a classic model used to test oxidative stress susceptibility or antioxidant efficiency in the RPE cell [18,31,32]. The present study demonstrates that treating ARPE-19 cells with H_2_O_2_ results in a marked loss of viability. However, treatment with AST decreased the cell viability loss significantly. ROS-mediated cellular damage was greatly reduced with AST pretreatment in retinal ganglion cells, human neuroblastoma cells, and human lymphocytes [8,33,34]. In this study, treatment with AST resulted in much lower levels of H_2_O_2_-induced intracellular production of ROS. The results of this study, as well as many earlier studies, suggest that AST has a direct antioxidant effect by scavenging ROS from the environment [8,33,34]. Moreover, flow cytometry showed that H_2_O_2_-induced cell apoptosis in ARPE-19 cells is greatly reduced by treatment with AST. We examined the expression of cleaved caspase-3, which is known to be a stimulator of apoptosis, to further explore the effect of AST on apoptotic cell death caused by exposure to H_2_O_2_. In the present study, treatment with AST significantly reduced the expression of apoptotic protein cleaved caspase-3 induced by H_2_O_2_. These results indicate that AST protects ARPE-19 cells from H_2_O_2_-induced cell damage via its antiapoptotic and antioxidative effects.

We investigated the potential pathway involved in the protective effect of AST against oxidative stress in ARPE-19 cells. In recent years, the Nrf2-ARE pathway has been characterized as an important endogenous mechanism that attenuates oxidative stress [35,36]. Nrf2 is an obligate transcription factor that could bind to the ARE to induce the expression of Phase II enzymes [37]. In the absence of oxidant damage, Nrf2 interacts with the chaperone Keap1, whereas in an oxidant environment, Nrf2 dissociates from Keap1, activated Nrf2 translocates to the nucleus and binds to the ARE, and then Phase II enzymes are expressed [38]. NQO1 reduces quinones via a two-electron reduction, limiting the subsequent generation of ROS [39,40]. HO-1 catalyzes the rate-limiting step in heme catabolism, resulting in formation of the antioxidant bilirubin when biliverdin reductase is present [41]. GCL controls the production of glutathione, which is the most abundant endogenous antioxidant thiol [37]. The GCL holoenzyme is a heterodimer consisting of GCLC and GCLM [42]. Earlier studies indicated that activation of the Nrf2-ARE pathway and increased expression of the following Phase II enzymes could protect RPE cells against oxidative damage [37,43]. Earlier studies showed that AST and other carotenoids can activate the Nrf2-ARE pathway in cancer cells and in the rat liver [44,45]. In our study, we investigated whether AST, a well-known potent antioxidant, can increase Nrf2 nuclear localization and promote the expression of Phase II enzymes in ARPE-19 cells. As we have shown, treatment with AST can increase the nuclear localization of Nrf2 and consequently increase the expression of Phase II enzymes regulated by Nrf2, including NQO1, GCLC, GCLM, and HO-1. As shown in our results, treatment with AST effectively protects ARPE-19 cells from H_2_O_2_-induced decreases in cell viability and inhibits cell apoptosis and the intracellular production of ROS. All of these protective effects probably arise from the enhancement of the Phase II antioxidant enzyme system.

Although there is more than one pathway AST could activate, including ERK, NF-κB, and PI3K/Akt [22,29,46], we focused on Akt because it has a key role in regulating many important proteins involved in cell survival through profound antioxidant and antiapoptotic actions [4]. Earlier studies demonstrated that adding H_2_O_2_ to RPE cells increased intracellular ROS production significantly and, simultaneously, caused detectable Akt activation [21]. In the present study, Akt phosphorylation was moderately enhanced in the stimulation of H_2_O_2_. Moreover, 20 μΜ AST notably increased Akt phosphorylation in ARPE-19 cells, and this effect was abolished when the cells were treated with the highly specific Akt inhibitor LY294002. These results suggest that activation of the PI3K/Akt pathway is involved in the protection of AST against H_2_O_2_-induced oxidative stress in ARPE-19 cells.

**Figure 8:**
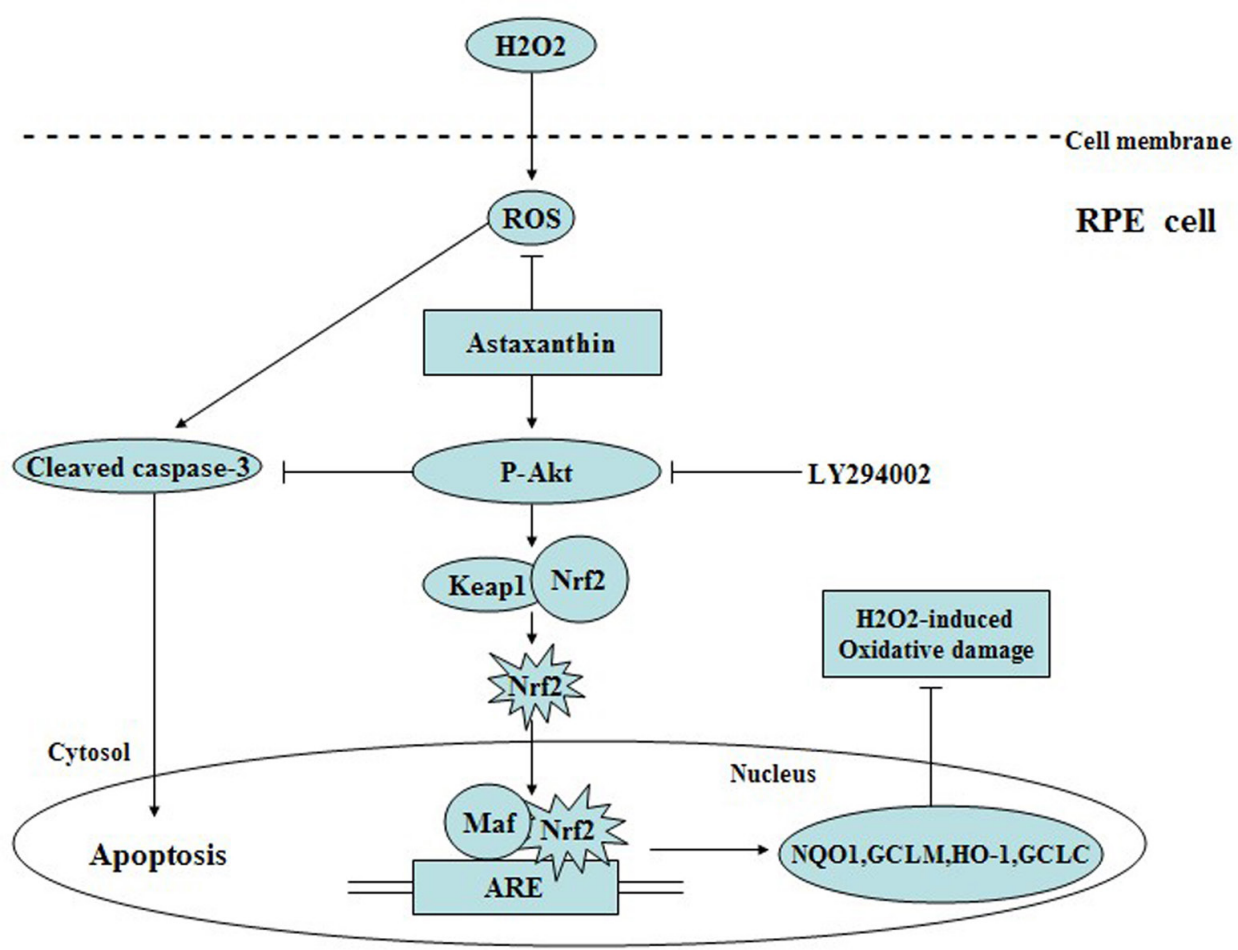
Schematic pathway diagram of the mechanism of astaxanthin in preventing hydrogen peroxide–induced oxidative stress in ARPE-19 cells.

The PI3K/Akt pathway has crucial roles in modulating Nrf2-ARE-dependent protection against oxidative stress in the RPE cell [20]. Our findings indicate that the increased nuclear localization of Nrf2 induced by AST was dependent on the activation of Akt, because LY294002 decreased the AST-induced enhancement of the nuclear localization of Nrf2 via inhibition of Akt phosphorylation. Furthermore, the results of our study demonstrate that the inhibitory effect of AST on H_2_O_2_-induced cleaved caspase-3 expression was partially reversed by LY294002. Our previous study indicated that LY294002 inhibited the AST-induced cytoprotective effect on cell viability and apoptosis against H_2_O_2_ (see the supplementary data). These results suggest that AST enhanced the Phase II antioxidant enzyme system and inhibited cell damage induced by H_2_O_2_ through activation of the PI3K/Akt pathway (Figure 8). The results of this study indicate that AST can offer a practical approach to the oxidative damage induced by H_2_O_2_ in ARPE-19 cells, which merits further investigation.

Taken together, the results of the present study provide strong evidence that treatment with AST attenuated the H_2_O_2_-induced oxidative stress in ARPE-19 cells and the protective mechanism was associated, at least in part, with the activation of the Nrf2-ARE and PI3K/Akt pathways. Other recent studies showed that AST protected retinal ganglion cells against H_2_O_2_-induced cell death [34]. These results are noteworthy and indicate that AST might be capable of protecting RPE cells and retinal neurons from oxidative damage. The results also suggest the possibility that administering AST is a potential therapeutic strategy for the prevention and therapy of AMD and other retinal disorders associated with oxidative stress.

## Appendix 1. LY294002 inhibited the AST-induced cytoprotective effect on cell viability against H2O2 in ARPE-19 cells.

To access the data, click or select the words “Appendix 1.” The ARPE-19 cells were treated with different concentrations of AST (0, 5, 10 and 20 µM) for 24 h and then treated with or without 10 µM LY294002 for 30 min before incubation with or without 200 µM H2O2 for 24 h. Data are shown as mean ± SD (n=6); *P <0.05 vs control. In all cases, the control is untreated RPE cells. # P <0.05 vs H2O2-induced cells without treatment with AST; §P<0.05 vs H2O2 + 20 µM AST.

## Appendix 2. LY294002 inhibited the AST-induced cytoprotective effect on cell apoptosis against H2O2 in ARPE-19 cells.

To access the data, click or select the words “Appendix 2.” (A) The ARPE-19 cells were incubated with different concentrations of AST (10 µM and 20 µM) for 24 h and then treated with or without 10 µM LY294002 for 30 min before incubation with or without 200 µM H2O2 for 24 h. Flow cytometry recording shows the apoptosis rate of ARPE-19 cells. (B) Summarized data showing the rate of apoptotic cells detected by ﬂow cytometry. Data are shown as mean ± SD (n=6); *P <0.05 vs control; # P <0.05 vs H2O2-induced cells without treatment with AST; §P<0.05 vs H2O2 + 20 µM AST.
